# Supplementary material for: Impact of Exercise-Induced Pulmonary Hypertension on Right Ventricular Function and on Worsening of Cardiovascular Risk in HIV Patients
Source: J Clin Med. 2022 Dec 10;11(24):7349. doi: 10.3390/jcm11247349 (PMC9781486; doi:10.3390/jcm11247349)
Supplement: Supplementary file 1 [file jcm-11-07349-s001.zip › jcm-2028709-supplementary.pdf]

**Online Table S1. Demographics, medical history and medication use according to presence and absence of ExPH at CPET**				
**Clinical parameter**	**ExPH at CPET**	**mean**	**sd**	**p-value**
Age	*no*	56,21	9,76	0,615
	yes	52,38	13,86	
BSA	*no*	1,91	0,20	0,475
	yes	1,86	0,24	
SAP	*no*	126,36	12,20	0,652
	yes	124,62	10,50	
DAP	*no*	74,24	7,08	0,745
	yes	75,00	7,07	
		**ExPH at CPET**		
		**no**	**yes**	
Gender	F	11	5	0,742
	M	22	8	
Smoke	no	16	3	0,264
	yes	16	9	
Functional class FC-WHO	I	32	3	<0,001
	II	1	0	
	III	0	10	
Chronic liver disease	*no*	16	8	0,425
	yes	17	5	
Kidney disease	*no*	29	12	0,664
	yes	4	1	
Tyroid disease	*no*	29	11	0,767
	yes	4	2	
Arterial hypertension	*no*	29	13	0,189
	yes	4	0	
Dislipidemia	*no*	10	6	0,309
	yes	23	7	
Diabetes mellitus	*no*	29	11	0,767
	yes	4	2	
Familiarity for CAD	*no*	26	10	0,891
	yes	7	3	
Calcium channel blockers	*no*	29	12	0,206
	yes	4	0	
Hypoglycaemic drugs	*no*	10	6	0,309
	yes	23	7	
Beta blockers	*no*	32	13	0,526
	yes	1	0	
ACE inhibitors or sartanics	*no*	32	13	0,526
	yes	1	0	
Tyroid hormones	*no*	29	11	0,767
	yes	4	2	
Lipid lowering drugs	*no*	10	6	0,309
	yes	23	7	

Abbreviations: ExPH, isolated exercise pulmonary hypertension; CPET, cardiopulmonary exercise test; BSA, body surface area; SAP, systolic arterial pressure; DAP, diastolic arterial pressure; CAD, coronary artery disease; F, female; M, male; ACE, angiotensin converting enzyme
